# Assessment of Health Status in Patients with Newly Diagnosed Chronic Obstructive Pulmonary Disease

**DOI:** 10.1371/journal.pone.0082782

**Published:** 2013-12-09

**Authors:** Yongping Gao, Qi Hou, Haoyan Wang

**Affiliations:** 1 Department of Respiratory Medicine, Beijing Friendship Hospital, Capital Medical University, Beijing, China; 2 Department of Pharmacology, Institute of Materia Medica, Peking Union Medical College and Chinese Academy of Medical Sciences, Beijing, China; University of North Carolina at Chapel Hill, United States of America

## Abstract

**Subject:**

Chronic obstructive pulmonary disease (COPD) is a common disease worldwide. This study aimed to investigate the health status of patients with newly diagnosed COPD.

**Methods:**

A total of 45 healthy controls and 218 patients with newly diagnosed COPD were recruited. Pulmonary function test (PFT) values, COPD assessment test (CAT) scores, exacerbation history, and demographics were recorded.

**Results:**

Forced expiratory volume in 1 s percent (FEV1%) predicted was significantly decreased and the CAT score was significantly increased in patients with COPD compared with healthy controls (*P* <0.001). Among the COPD patients, the most commonly reported respiratory symptoms were cough (86.7%), sputum (80.3%), and dyspnea (45%). A total of 86.2% patients were in the moderate or severe stage (spirometric classification) of COPD, and 71.5% were in Group C or Group D (combined assessment). A total of 33.9% of the patients had 2 or more exacerbations in the previous year. Nearly half of the patients (45.4%) had a high CAT score of ≥10. Patients with a history of more exacerbations had a higher CAT score.

**Conclusions:**

Most COPD patients were symptomatic and appeared to have moderate to severe airflow limitation or a high risk of exacerbation before definitely being diagnosed with COPD using the PFT.

## Introduction

Chronic obstructive pulmonary disease (COPD) is a major cause of chronic morbidity and mortality worldwide [1], and is predicted to become the third leading cause of death in the world by 2020 [2]. Early detection and preventive measures may reduce both morbidity and mortality because COPD is a preventable and treatable disease characterized by persistent airflow limitation. Corticosteroids combined with long acting β2-adrenoceptor agonists improve lung function and quality of life, prevent exacerbation, and reduce hospitalization [3,4]. However, the prevalence and under-diagnosis of COPD in adult patients in the primary care setting are high [5,6]. Many people have this disease for years, and die prematurely from it or its complications. Spirometry is the most objective measurement of airflow limitation to determine the severity of COPD. The COPD assessment test (CAT) is recommended for assessing symptoms, with a CAT score of ≥10 indicating a high level of symptoms. Exacerbations of COPD are important events in the course of the disease because they contribute to the overall severity in individual patients. 

This study aimed to investigate the health status of patients with newly diagnosed COPD, including pulmonary function, symptoms, and exacerbation history.

## Methods

### Study population and design

From March 2011 to March 2012, we analyzed 218 patients who underwent spirometry and were newly diagnosed with COPD. A total of 45 healthy controls were also enrolled. The control subjects were chosen from visitors to the hospital for healthy examination with approximately the same age as the patients. First, all of the subjects underwent spirometry with a bronchodilator test. Pulmonary function test (PFT) was performed using the acceptability standards outlined by the American Thoracic Society [7]. Patients were recruited if they met the established diagnostic criteria [8]. None of these patients had previously been diagnosed with COPD or other chronic respiratory diseases. When all the subjects were included in the study, a detailed medical history was compiled with a designed questionnaire. The data collected included age, sex, weight, height, tobacco habit, symptoms (cough, sputum, and dyspnea), exacerbation history, presence of chronic associated comorbidities (cardiovascular disease, hypertension, and diabetes mellitus), symptoms evaluated according to CAT, and pharmacological treatments related to COPD. 

### Ethical aspects

The study was approved by Beijing Friendship Hospital, Capital Medical University (Beijing, China). All of the patients were informed of the characteristics and objectives of the study, and gave their written consent for participation.

### Statistical analysis

Statistical analysis was performed with SPSS 18.0 (IBM). The results of the continuous variables are presented by mean and standard deviation. The Student’s t-test was used to compare data with normal distribution and the Mann–Whitney U test was used if the distribution was not normal. Chi-square tests were used for categorical variables. The relationship between two related values was assessed by Pearson’s correlation. Results were considered statistically significant when the P value was <0.05.

## Results

Forced expiratory volume in 1 s percent (FEV1%) predicted was significantly decreased and the CAT score was significantly increased in patients with COPD compared with healthy controls (*P* <0.001). Among the 218 patients with COPD, 194 patients (89%) were department of respiratory medicine outpatients and inpatients, and 24 patients (11%) were from other internal medicine departments. None of the patients received inhaled corticosteroids or bronchodilators before being enrolled. A total of 65% of the study population were current smokers and 8% were never smokers. The most commonly reported respiratory symptoms were cough (86.7%), sputum (80.3%), and dyspnea (45%). The demographics and characteristics of the patients are shown in Table 1.

**Table 1:** Clinical characteristics of patients with COPD and healthy control subjects.

	Healthy Controls	COPD	P
Gender M|F	40|5	183|35	0.499
Age, year	67.1(8.7)	66.7(7.4)	0.887
Pack years	7.5(5.6)	35.0(23.5)	<0.001
Smoking, Never|Ex|Current	7|2|36	18|59|141	<0.001
FEV1 % predicted, post bronchodilatation	98.05(11.2)	47.34(14.25)	<0.001
FEV1/FVC %, post bronchodilatation	78.21(5.32)	48.09(11.94)	<0.001
CAT	4(2.1)	10(5.3)	<0.001
Exacerbation history	0.07(0.25)	1.1(1.14)	<0.001
Cough (n, %)	3(6.7)	189(86.7)	<0.001
Sputum (n, %)	2(4.4)	175(80.3)	<0.001
Dyspnea (n, %)	1(2.2)	98(45.0)	<0.001

Notes: Number of the patients is expressed in absolute numbers and percentage of the sample. The remaining values are expressed by mean and standard deviation.

Abbreviations: FEV1, forced expiratory volume in 1 second; FVC, forced vital capacity; CAT, COPD assessment test.

The patients were stratified by CAT scores (Table 2) and frequency of exacerbations (Table 3). FEV1% predicted was not different between the groups. It is close to significance that those with a lower CAT score had a lower FEV1 (*P* = 0.071). The number of patients with a CAT score of ≥10 was similar to that of patients with a score of <10 (99 vs. 119). Seventy-four patients (33.9%) had 2 or more exacerbations in the previous year. Patients with a history of more exacerbations had a significantly higher CAT score compared with those with a history of fewer than 2 (*P* = 0.021). The relationship between the CAT score and the frequency of exacerbations was further analyzed using Pearson’s correlation. There was a weak correlation between exacerbation history and the CAT score (*r* = 0.271, *P* = 0.011).

**Table 2:** Characteristics of COPD patients stratified by CAT scores.

	CAT<10	CAT≥10	P
N (%)	119(54.6)	99(45.4)	/
Age, year	65.7(8.1)	68.3(7.7)	0.125
Pack years	40.3(23.3)	30.8(24.7)	0.067
FEV1, % predicted post bronchodilatation	44.6(13.5)	50.5(16.7)	0.071

Notes: Number of the patients is expressed in absolute numbers and percentage of the sample. The remaining values are expressed by mean and standard deviation.

Abbreviations: FEV1, forced expiratory volume in 1 second; CAT, COPD assessment test.

**Table 3:** Characteristics of COPD patients stratified by the frequency of exacerbations.

	Exacerbation history <2	Exacerbation history≥2	P
N (%)	144(66.1)	74(33.9)	/
Age, year	66.6(8.1)	67.3(7.6)	0.670
Pack-years	34.5(21.0)	38.8(29.8)	0.427
FEV1, % predicted post bronchodilatation	47.9(15.7)	46.3(14.6)	0.649
CAT	8.8(5.9)	12.0(6.3)	0.021

Notes: Number of the patients is expressed in absolute numbers and percentage of the sample. The remaining values are expressed by mean and standard deviation.

Abbreviations: FEV1, forced expiratory volume in 1 second; CAT, COPD assessment test.

**Figure 1:**
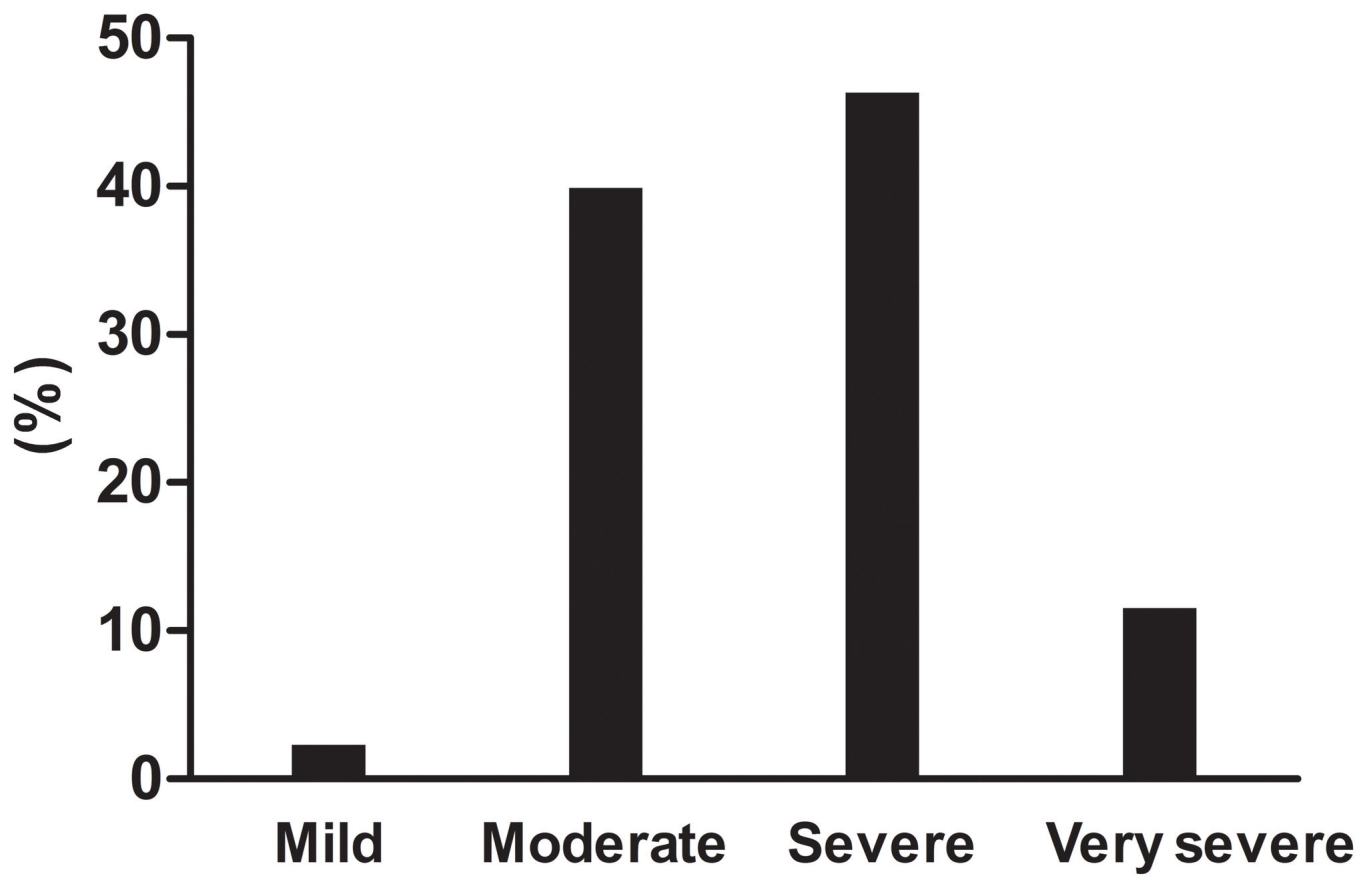
Distribution of the severity stages of COPD according to airway obstruction. Analysis of the severity of airway obstruction in patients with COPD showed that the prevalence of moderate and severe COPD was 39.9% and 46.3%.

**Figure 2:**
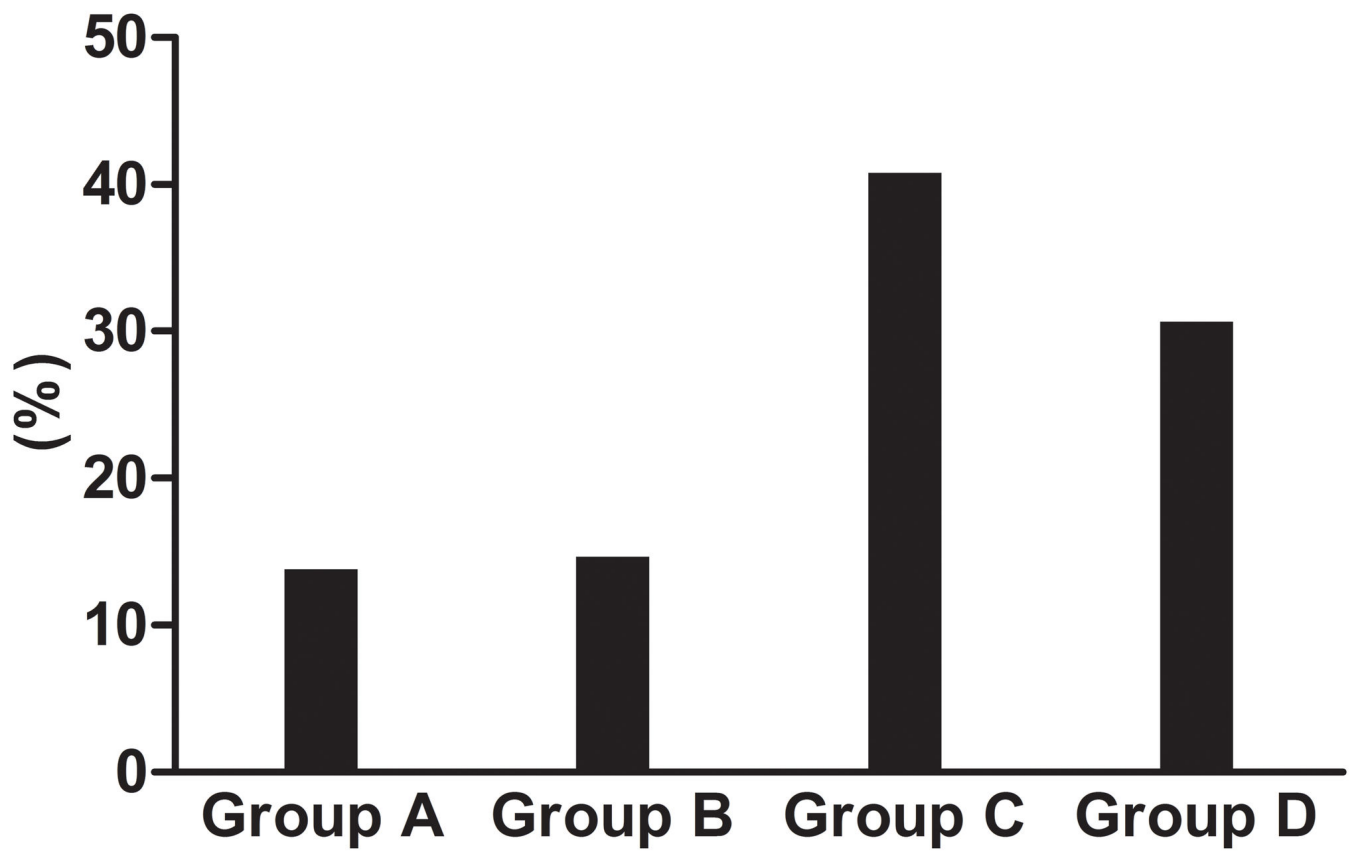
Distribution of the severity stages of COPD according to combined assessment. Patients were assessed with symptoms (CAT), spirometric classification, and exacerbation history. The prevalence of Group C and Group D was 40.8% and 30.7%.

Analysis of the severity of airway obstruction in patients with COPD showed that the prevalence of moderate to severe COPD with FEV1% predicted between 30% and 80% was 86.2% (Figure 1). Patients were further assessed with symptoms (CAT), spirometric classification, and exacerbation history according to GOLD 2011[8]. The prevalence of Group C and Group D was 40.8% and 30.7% (Figure 2). 

## Discussion

This study represents the first attempt to assess the health status of inpatients and outpatients with newly diagnosed COPD. In the present study, FEV1% predicted was significantly decreased and the CAT score was significantly increased in patients with COPD compared with healthy controls. Most of the patients had chronic symptoms with cough, sputum and/or dyspnea before the first PFT. Nearly half of the patients had a high CAT score of ≥10, indicating a high level of symptoms. We also found that one-third of the newly diagnosed patients had 2 or more exacerbations in the preceding year, indicating a high risk of exacerbation. These results showed that the majority of patients had a poor health status before definitely being diagnosed with COPD. Large numbers of newly detected patients were symptomatic and needed treatment. Forced vital capacity testing was introduced in China in the early 20th century. In 2002, Zheng Jinping [9] investigated the clinical application of the PFT with a questionnaire survey performed in 212 hospitals covering 29 provinces and found that application of the PFT was not popular, and varied from hospital to hospital in China. Currently, an increasing amount of physicians are recognizing the importance of the PFT, but not all of them have a correct understanding of its significance. Most patients with chronic symptoms are treated in community hospitals or clinics without the PFT or further diagnosis in China. The rate of missed diagnosis phenomenon in inpatients is also high [10]. Further work is required to enhance recognition of the harm of COPD and the importance of the PFT for early diagnosis. 

We also found that most of the patients were in the moderate or severe stage of COPD according to airflow limitation and in Group C or D, indicating a high risk of exacerbation. A previous study showed that 79% of patients with newly diagnosed COPD had mild to moderate airway obstruction [11]. This previous result was optimistic because the enrolled subjects were volunteers, while in the present study, outpatients or inpatients were studied. 

In addition, our study showed that patients with a history of more exacerbations had a higher CAT score. There was a weak correlation between the frequency of exacerbation and the CAT score. This result is consistent with previous reports [12,13]. The current study also showed that FEV1% predicted was not different among the different CAT score groups. Although not statistically significant, it is close to significance that those with a lower CAT score actually had a lower FEV1. There are several validated questionnaires to assess symptoms and quality of life in patients with COPD. Previous studies have described the relationship between quality of life and pulmonary function. Jones PW [14] reported that health status scores were weakly correlated with FEV1. According to GOLD, there is also a weak correlation between FEV1, symptoms, and impairment of a patient’s healthy quality of life. In contrast, an early study reported FEV1 was not associated with the Medical Research Council grade [15]. Quality of life is unique to the individual and is affected by many factors, which may partially explain the differences among studies. Further study need to be done on the relationship between quality of life and pulmonary function.

The current study has limitations that should be considered when interpreting the results. The sample was small and the study did not involve multiple centers. Our results need to be confirmed by a large sample and a multi-center investigation. 

## Conclusions

Most COPD patients have a poor health status and have a history of chronic symptoms before definitely being diagnosed with COPD by the PFT. Patients’ and clinicians’ awareness of COPD should be improved to make an early diagnosis. 
